# ATP-sensitive potassium channels alter glycolytic flux to modulate cortical activity and sleep

**DOI:** 10.1073/pnas.2416578122

**Published:** 2025-02-18

**Authors:** Nicholas J. Constantino, Caitlin M. Carroll, Holden C. Williams, Hemendra J. Vekaria, Carla M. Yuede, Kai Saito, Patrick W. Sheehan, J. Andy Snipes, Marcus E. Raichle, Erik S. Musiek, Patrick G. Sullivan, Josh M. Morganti, Lance A. Johnson, Shannon L. Macauley

**Affiliations:** ^a^Department of Physiology, University of Kentucky, Lexington, KY 40508; ^b^Department of Neuroscience, University of Kentucky, Lexington, KY 40508; ^c^Sanders Brown Center on Aging, University of Kentucky, Lexington, KY 40508; ^d^Department of Psychiatry, Wake Forest School of Medicine, Winston-Salem, NC 27101; ^e^Spinal Cord and Brain Injury Research Center, University of Kentucky, Lexington, KY 40508; ^f^Department of Psychiatry, Washington University School of Medicine, St. Louis, MO 63110; ^g^Department of Neurology, Washington University School of Medicine, St. Louis, MO 63110; ^h^Department of Radiology, Washington University School of Medicine, St. Louis, MO 63110; ^i^Department of Neuroscience, Washington University School of Medicine, St. Louis, MO 63110; ^j^Department of Psychology & Brain Sciences, Washington University, St. Louis, MO 63110; ^k^Department of Biomedical Engineering, Washington University School of Medicine, St. Louis, MO 63110

**Keywords:** K_ATP_ channels, metabolism, excitability, sleep, arousal

## Abstract

Metabolism plays a key role in the maintenance of sleep/wake states. Brain lactate fluctuations are a biomarker of sleep/wake transitions, where increased interstitial fluid (ISF) lactate levels are associated with wakefulness and decreased ISF lactate is required for sleep. ATP-sensitive potassium (K_ATP_) channels couple glucose-lactate metabolism with excitability. Using mice lacking K_ATP_ channel activity (e.g., Kir6.2^−/−^ mice), we explored how changes in glucose utilization affect cortical electroencephalography (EEG) activity and sleep/wake homeostasis. In the brain, Kir6.2^−/−^ mice shunt glucose toward glycolysis, reducing neurotransmitter biosynthesis and dampening cortical EEG activity. Kir6.2^−/−^ mice spent more time awake at the onset of the light period due to altered ISF lactate dynamics. Together, we show that Kir6.2-K_ATP_ channels act as metabolic sensors to gate arousal by maintaining the metabolic stability of sleep/wake states and providing the metabolic flexibility to transition between states.

## Significance

Glucose is not only an energy source for the brain, but also a biosynthetic substrate for neurotransmitter synthesis. Changes in glycolytic flux can alter neurotransmitter synthesis to reduce cortical electroencephalography (EEG) activity, arousal, and sleep despite sufficient energy availability. Kir6.2-K_ATP_ channels are metabolic sensors under circadian control that gate glycolytic flux, arousal, and sleep/wake transitions. The regulation of glycolytic flux by Kir6.2-K_ATP_ channels facilitates the maintenance of and transition between sleep/wake states.

Metabolism is intrinsically and bidirectionally linked to sleep/wake states (e.g., wake, NREM, REM) (1–4). Fluctuations in brain lactate levels are a biomarker of sleep/wake states, where interstitial fluid (ISF) lactate rises with the onset of wake and drops with NREM sleep (3, 5–7). Heightened arousal and complex behavioral states are strongly associated with lactate production more than oxidative metabolism (1, 2, 8). Conversely, ISF lactate levels must drop to transition to and sustain NREM or REM sleep (7). While cerebral metabolism is associated with both sleep/wake cycles and circadian rhythms, ISF lactate fluctuations are associated more with sleep/wake transitions than ISF glucose (7). Altogether, this suggests changes in cellular metabolism can impact sleep/wake states and arousal; however, the molecular mechanisms governing this relationship are not fully understood.

One mechanism that couples metabolism with cellular activity is ATP-sensitive potassium (K_ATP_) channels. When ATP levels are high, K_ATP_ channels close, biasing cells toward excitability. Conversely, when ATP levels are low, the channels open, hyperpolarizing the cell, and limiting excitability. When the expression or function of K_ATP_ channels is altered, we hypothesize that this impairs a cell’s ability to sense and utilize glucose, which feeds forward to impair cellular excitability, physiology, and behavior.

K_ATP_ channels are composed of 4 pore forming subunits (e.g., Kir6.1, Kir6.2) and 4 sulfonylurea regulatory receptors (e.g., Sur1, Sur2A/B). Within the brain, K_ATP_ channels containing the Kir6.2 and Sur1 subunits (Kir6.2-K_ATP_ channels) are largely found on excitatory and inhibitory neurons, where they act as metabolic sensors to regulate excitability (9–14). One way Kir6.2-K_ATP_ channels sustain neuronal firing is by coupling changes in ISF glucose with ISF lactate (7, 10). We previously demonstrated that loss of Kir6.2-K_ATP_ channels uncoupled the relationship between ISF glucose and ISF lactate (10), suggesting changes in glucose utilization or glycolytic flux. While Kir6.2-K_ATP_ channels are found throughout the brain, K_ATP_ channels are enriched in glucose sensing (15) and orexinergic neurons (16) within the hypothalamus (17), suggesting a role in sleep/wake homeostasis, circadian rhythms, and arousal (8, 18–22). Therefore, we hypothesized that K_ATP_ channel deletion would dampen cortical EEG activity and disrupt the brain’s diurnal and sleep/wake rhythms by altering glycolytic flux and lactate dynamics.

Using mice which lack Kir6.2-K_ATP_ channel activity globally (e.g., Kir6.2^−/−^ mice), we investigated whether Kir6.2-K_ATP_ channel deletion impacted the relationship between metabolism, excitability, and sleep. We used stable isotope resolved metabolomics (SIRM) to explore shifts U-^13^C-glucose utilization in the brains of mice lacking Kir6.2-K_ATP_ channel activity. Then, we examined whether changes in glucose utilization were driven by primary deficits in synaptic or nonsynaptic mitochondria rather than changes in metabolic sensing or glycolytic flux. Next, we used cortical EEG/EMG and behavioral assays to investigate whether Kir6.2-K_ATP_ channel deletion impacted EEG power and its relationship with arousal, anxiety, and cognition. Finally, using simultaneous EEG/EMG recordings with intrahippocampal monitoring of ISF glucose and lactate levels, we explored how K_ATP_ channel deletion affected sleep/wake homeostasis relative to ISF lactate dynamics. Together, our data show that Kir6.2-K_ATP_ channels act as metabolic sensors to gate glycolytic flux, which impacts cortical EEG activity, arousal, and sleep/wake homeostasis. This suggests the brain senses changes in energy needs through Kir6.2-K_ATP_ channel activity, which in turn affects the brain’s ability to maintain and transition between sleep/wake states. It further suggests that alterations in the expression or function of K_ATP_ channels impair the cell’s ability to detect and utilize glucose to regulate cellular excitability and sleep. These studies highlight the importance of metabolic flexibility to maintain arousal, biological rhythms, and sleep/wake homeostasis.

## Results

### Kir6.2-K_ATP_ Channels Are Predominantly Located on Excitatory and Inhibitory Neurons Within the Brain.

**Fig. 1.**
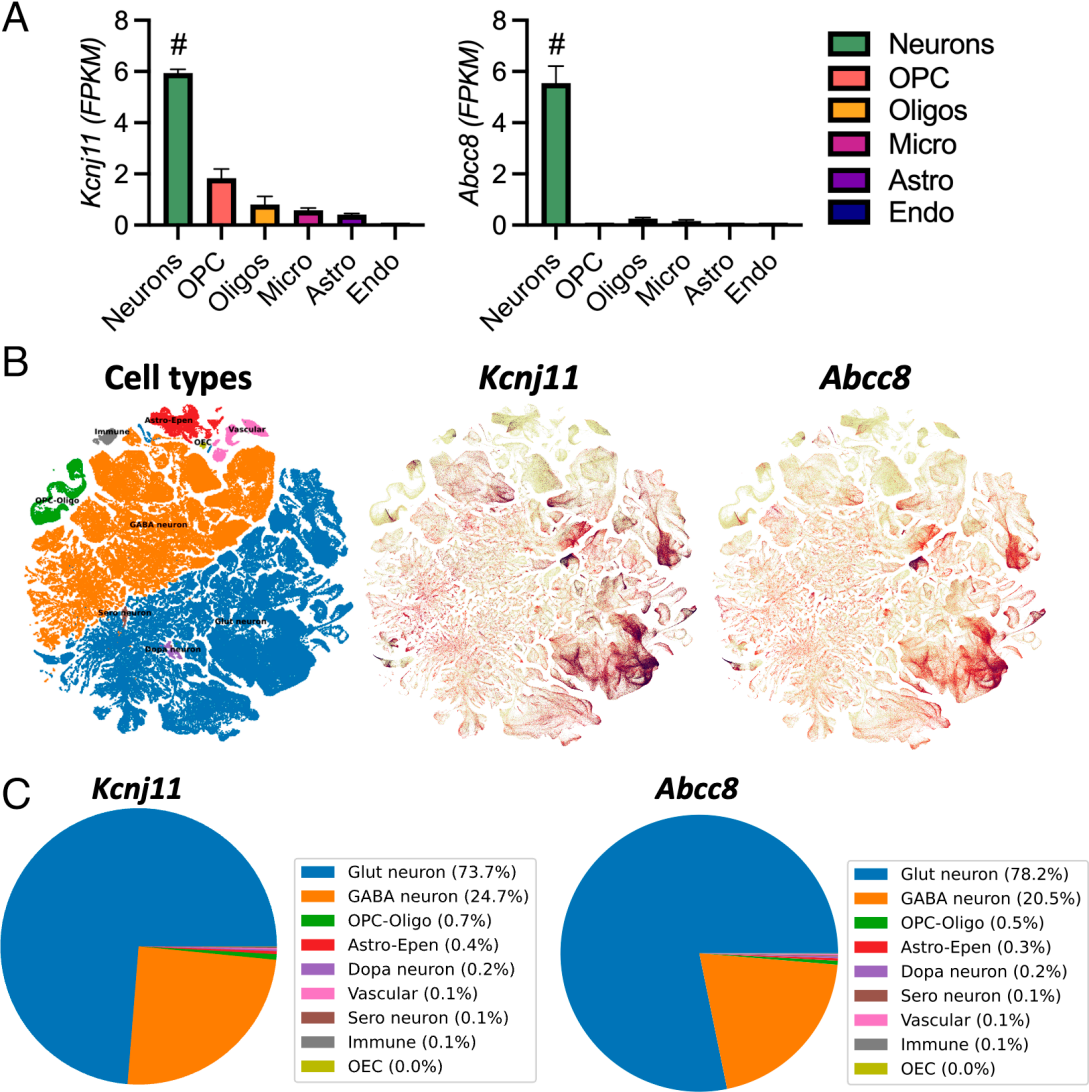
Kir6.2-K_ATP_ channels are expressed on excitatory and inhibitory neurons within the mouse brain. (*A*) Expression of Kir6.2-K_ATP_ channel subunits within the brain (*Kcnj11* and *Abcc8*) (brainrnaseq.org). *Kcnj11* and *Abcc8* are primarily expressed on neurons, not glia or vascular cells (*P* < 0.0001, one-way ANOVA). (*B*) UMAP plot of cell type designations, *Kcnj11* expression, and *Abcc8* expression (portal.brain-map.org/atlases-and-data/bkp/abc-atlas). Expression was predominantly localized to glutamatergic and GABAergic neurons. (*C*) Pie chart of percentage of transcripts of *Kcnj11* and *Abcc8* in each cell type (portal.brain-map.org/atlases-and-data/bkp/abc-atlas). Data reported as ± SEM. n = 2 mice in brainrnaseq.org. # = *P* < 0.0001.

By leveraging publicly available singe cell RNA sequencing databases (brainrnaseq.org and portal.brain-map.org/atlases-and-data/bkp/abc-atlas) (23, 24), we expanded upon our previous work (10–12) demonstrating that Kir6.2-K_ATP_ channels in the brain are primarily located on neurons, not glia or vasculature (Fig. 1*A*, *P* < 0.0001). Moreover, *Kcnj11* and *Abcc8,* which code for the K_ATP_ channel subunits Kir6.2 and SUR1, respectively, are primarily found on excitatory and inhibitory neurons (Fig. 1 *B* and *C*), including glutamatergic (73.7% and 78.2%, respectively) and GABAergic neurons (24.7% and 20.5%, respectively) (24). Less than 2% of all transcripts are localized to nonneuronal cells in the brain, like glia and vascular cells (Fig. 1 *B* and *C* and *SI Appendix*, Fig. S1). This suggests that K_ATP_ channel deletion in the global Kir6.2 knockout (Kir6.2^−/−^) should have the greatest impact on neuronal metabolism and excitability.

### Kir6.2-K_ATP_ Deficient (Kir6.2^−/−^) Brains Increase Glycolysis at the Expense of Neurotransmitter Synthesis.

**Fig. 2.**
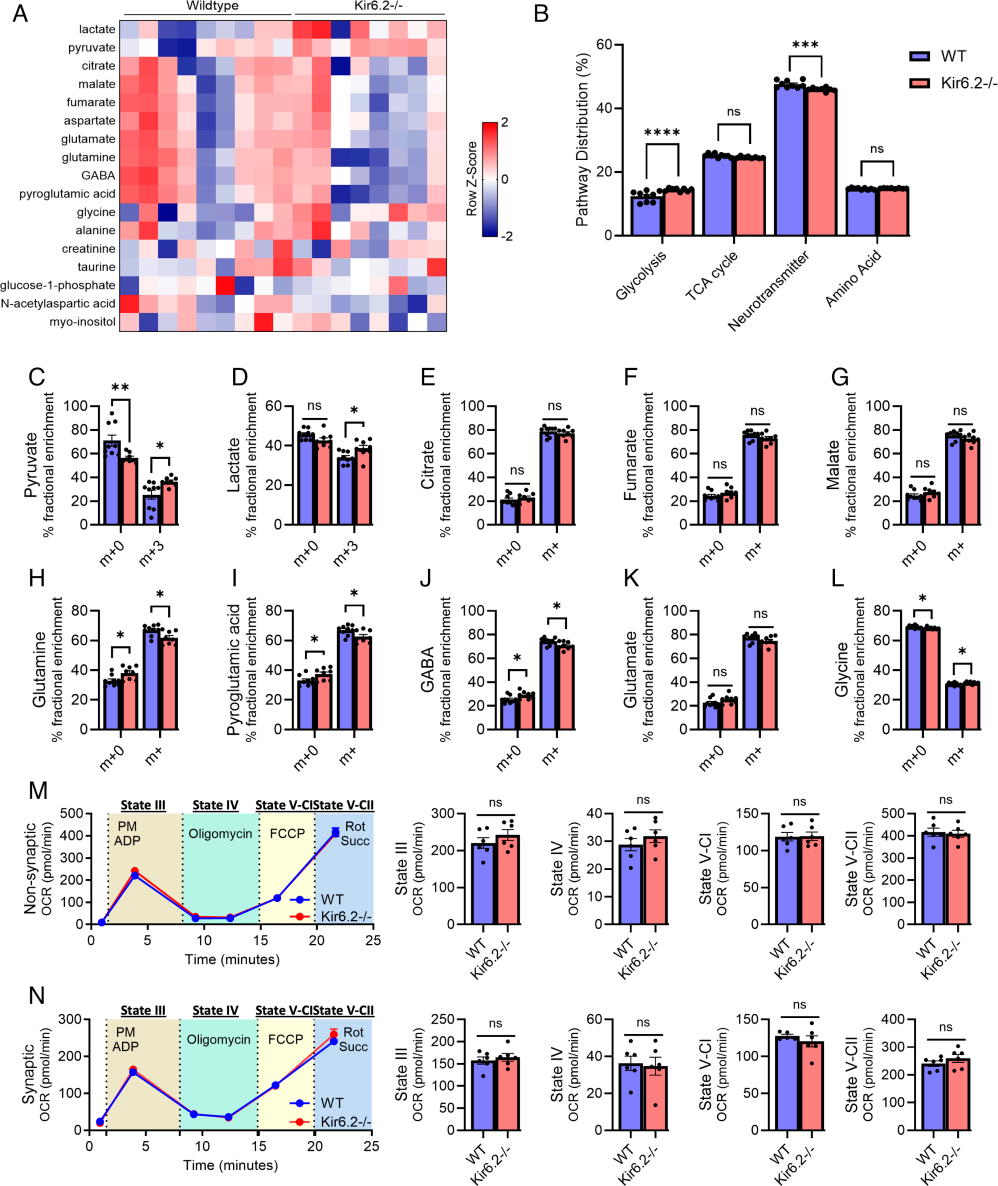
Kir6.2^−/−^ brains shunt glucose toward glycolysis and away from neurotransmitter synthesis, despite normal synaptic and nonsynaptic mitochondrial respiration. (*A*) Heatmap of metabolites following ^13^C-glucose administration. (*B*) Incorporation of ^13^C-glucose into metabolic pathways. Kir6.2^−/−^ mice shunt glucose toward glycolysis and away from neurotransmitter synthesis (*P* < 0.0001 and *P* = 0.0003, two-way ANOVA). (*C* and *D*) Unlabeled pyruvate is reduced, while fully labeled pyruvate and lactate are increased in Kir6.2^−/−^ mice (*P* < 0.01, *P* < 0.05, two-way ANOVA). (*E*–*G*) TCA intermediates citrate, fumarate, and malate are unaltered in Kir6.2^−/−^ mice (*P* > 0.1, two-way ANOVA). (*H*–*J*) Labeled glutamine, pyroglutamic acid, and GABA are reduced in Kir6.2^−/−^ mice (*P* < 0.05, two-way ANOVA), while unlabeled metabolites are elevated (*P* < 0.05, two-way ANOVA). (*K*) Glutamate is unaltered (*P* > 0.1, two-way ANOVA). (*L*) Labeled glycine is increased in Kir6.2^−/−^ mice (*P* < 0.05, two-way ANOVA), while unlabeled glycine is decreased (*P* < 0.05, two-way ANOVA). (*M*) Nonsynaptic mitochondrial oxygen consumption rates (OCR) are unchanged in Kir6.2^−/−^ mice (*P* > 0.1, unpaired *t* tests). (*N*) Synaptic mitochondrial OCR are unchanged in Kir6.2^−/−^ mice (*P* > 0.1, unpaired *t* tests). Data reported as ± SEM. n = 6 to 9 mice/genotype (*SI Appendix*, Fig. S1).

Following oral gavage of U-^13^C-glucose, cerebral glucose metabolism was quantified via stable isotope resolved metabolomics (SIRM) to assess glucose utilization in the Kir6.2^−/−^ and WT brain (25). A heatmap representation of relative abundance for each ^13^C-glucose labeled metabolite in Kir6.2^−/−^ and WT brains is shown in Fig. 2*A*. ^13^C-labeled metabolites were binned into pathways to explore patterns of glucose utilization more broadly in Kir6.2^−/−^ mice as previously described (25). For example, glutamate, glutamine, GABA, pyroglutamic acid, NAA, myo-inositol, and aspartate were binned as metabolites related to “neurotransmitter synthesis” due to their primary roles as neurotransmitters, their ability to modulate neurotransmitter signaling, or their role as neurotransmitter precursors in the brain (26–30). Pyruvate, lactate, and glucose-1-phosphate were binned into “glycolysis” because of their primary role in this metabolic pathway. Differences in glycolysis (pyruvate, lactate, glucose-1-phosphate) and neurotransmitter synthesis (glutamate, glutamine, GABA, pyroglutamic acid, NAA, myo-inositol, and aspartate) were observed in Kir6.2^−/−^ mice compared to WT (Fig. 2*B*, *P* < 0.0001, *P* < 0.0005), while metabolites associated with the TCA cycle (citrate, malate, fumarate) and amino acid synthesis (alanine, creatinine, taurine, glycine) were similar across groups (Fig. 2*B*, *P* = 0.1496, *P* = 0.7309). Increased glycolysis in Kir6.2^−/−^ was driven by increased labeling of pyruvate and lactate following a ^13^C-glucose challenge (Fig. 2 *C* and *D* and *P* < 0.05). The unlabeled and labeled pool of TCA intermediates were unchanged in Kir6.2^−/−^ mice (Fig. 2 *E*–*G*, *P* = 0.3853, *P* = 0.1949, *P* = 0.1739, respectively). Conversely, Kir6.2^−/−^ mice had decreased labeled glutamine and pyroglutamic acid (Fig. 2 *H* and *I*, *P* < 0.05), precursors to neurotransmitters glutamate and GABA (26, 27), suggesting reduced neurotransmitter synthesis from ^13^C-glucose. ^13^C-glucose labeled GABA was also decreased in Kir6.2^−/−^ mice (Fig. 2*J*, *P* < 0.05), while glutamate remained unchanged (Fig. 2*K*, *P* = 0.1292). Labeled glycine, an amino acid that acts as an inhibitory neurotransmitter and coagonist for NMDA receptor-mediated excitatory neurotransmission, was increased in Kir6.2^−/−^ mice (Fig. 2*L*, *P* < 0.05) (31, 32). Despite shifts in glucose utilization, the total abundance of most brain metabolites was similar across groups, with the exception of pyruvate which was elevated in Kir6.2^−/−^ mice (*SI Appendix*, Fig. S2, *P* < 0.05). This suggests in mice lacking K_ATP_ channel activity that glucose is preferentially utilized to fuel glycolysis at the expense of glucose-dependent neurotransmitter synthesis.

Next, we explored whether mice lacking Kir6.2-K_ATP_ channel activity have primary deficits in mitochondrial respiration which may explain glycolytic shifts and increased glycolysis. Here, we quantified mitochondrial respiration on synaptic (neuronal) and nonsynaptic (neuronal soma, glia, vascular cells) mitochondria from the Kir6.2^−/−^ and WT brains. No differences in OCR were found in Kir6.2^−/−^ mice compared to WT in either the synaptic or nonsynaptic pool (Fig. 2 *M* and *N*), confirming that mitochondrial respiration in the brain is unaltered with Kir6.2-K_ATP_ channel deficiency. Together, this suggests that increased glycolysis in the Kir6.2^−/−^ mice is not due to a primary mitochondrial defect, but due to changes in glycolytic flux caused by the loss of K_ATP_ channel activity.

### Kir6.2-K_ATP_ Channel Deletion Reduces Absolute EEG Power, Alpha and Theta Activity, and Arousal.

**Fig. 3.**
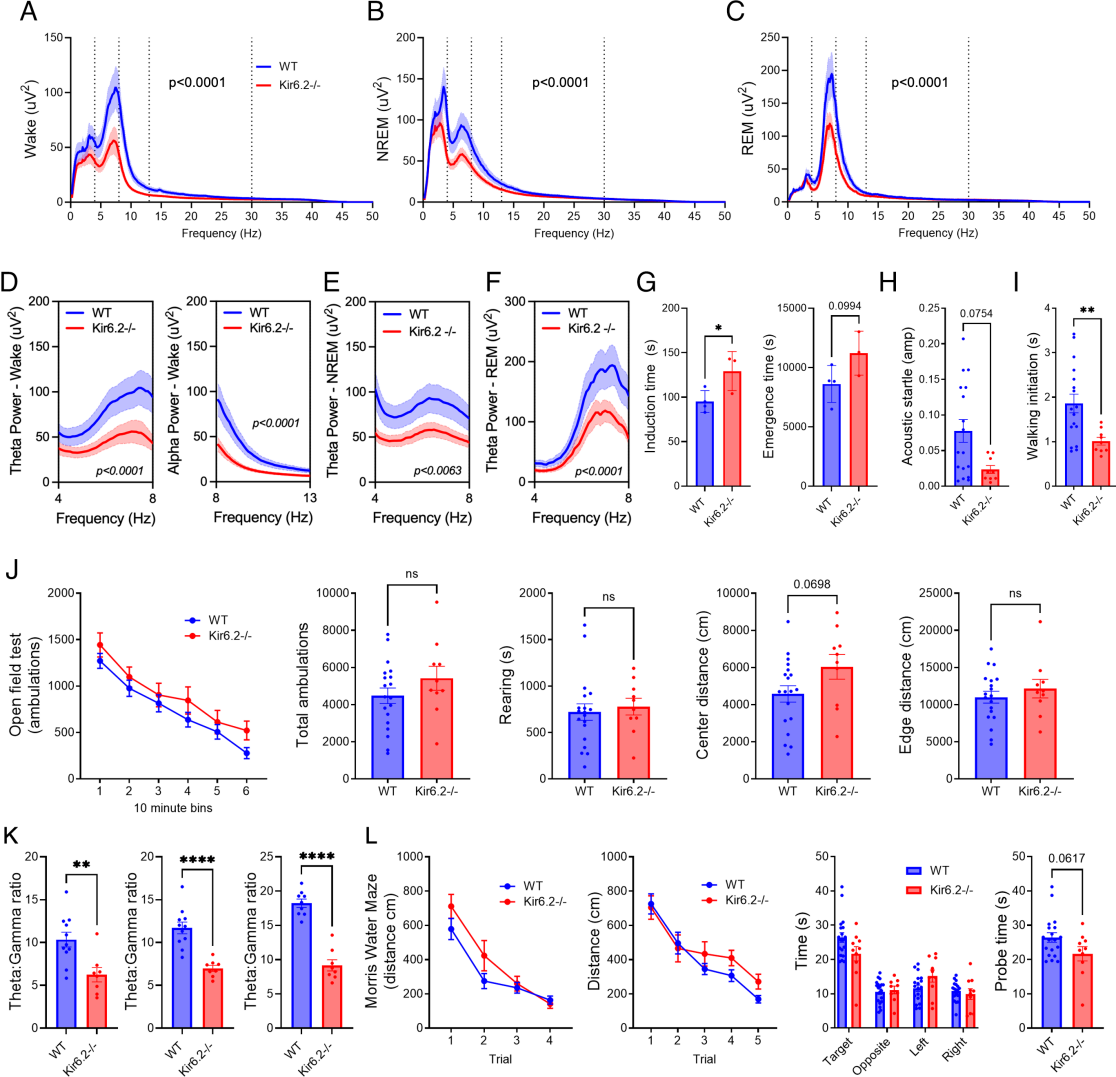
Reductions in cortical EEG power across sleep/wake states are reflected in changes in arousal, anxiety, and cognition. (*A*–*C*) Cortical EEG absolute power is decreased across wake, NREM, and REM in Kir6.2^−/−^ mice (*P* < 0.0001, two-way ANOVA). (*D*) In wake, absolute theta and alpha power are decreased in Kir6.2^−/−^ mice (*P* < 0.0001, two-way ANOVA). (*E* and *F*) In NREM and REM, absolute theta power is reduced in Kir6.2^−/−^ mice (*P* < 0.0063, *P* < 0.0001, two-way ANOVA). (*G*) During anesthesia challenges, induction and emergence time were increased in Kir6.2^−/−^ mice (*P* < 0.05, *P* = 0.0904, unpaired *t* test). (*H*) Kir6.2^−/−^ mice have reduced acoustic startle (*P* = 0.0754, unpaired *t* test). (*I*) Decreased latency to explore a novel environment in Kir6.2^−/−^ mice (*P* < 0.01, unpaired *t* test). (*J*) Activity is unaltered in Kir6.2^−/−^ mice, while distance traveled in the center of the chamber is elevated (*P* = 0.0698, unpaired *t* test). (*K*) Relative theta:gamma ratio is decreased in Kir6.2^−/−^ mice across states (*P* < 0.01, *P* < 0.0001, unpaired *t* test). (*L*) Kir6.2^−/−^ mice learned the Morris water maze (MWM) task, but spent less time in the target quadrant (*P* = 0.0617, unpaired *t* test). Data reported as means ± SEM. n = 8 to 11 mice/genotype for EEG/EMG, n = 10 to 20 mice/genotype for behavior.

Given the metabolomics data suggesting alterations in neurotransmitter synthesis, we explored whether Kir6.2^−/−^ mice have changes in neuronal activity and behavior. Cortical activity was recorded using skull screw EEG in Kir6.2^−/−^ and WT mice. Absolute power, a measure of the summative strength of cortical EEG activity, was dampened in Kir6.2^−/−^ mice across all frequencies and sleep/wake states (Fig. 3 *A*–*C*, *P* < 0.0001). This suggests that metabolic flux is associated with decreased EEG power in Kir6.2^−/−^ mice.

Absolute power was then binned into specific frequency bands, including delta (0.5 to 4 Hz), theta (4 to 8 Hz), alpha (8 to 13 Hz), beta (13 to 30 Hz), and gamma (30 to 50 Hz), to explore whether changes in absolute EEG power could predict alterations in behavior. Theta (4 to 8 Hz) and alpha (8 to 13 Hz) power, which are typically associated with arousal, sleep pressure, and state switching (33–37), were reduced in Kir6.2^−/−^ mice during wake (Fig. 3*D*, *P* < 0.0001). Theta band activity, which builds during NREM sleep and dominates EEG activity during REM sleep (37–40), decreased in both NREM and REM sleep in Kir6.2^−/−^ mice (Fig. 3 *E* and *F*, *P* < 0.0063, *P* < 0.0001).

Given the reductions in alpha and theta activity, we explored whether behaviors associated with arousal were affected in Kir6.2^−/−^ mice. First, we explored whether Kir6.2^−/−^ mice responded differently to anesthesia, as a proxy for how mice transition between consciousness or sleep/wake states (Fig. 3*G*). Induction time (e.g., wake to anesthetization) was increased while emergence time (e.g., anesthetization to wake) trended longer for Kir6.2^−/−^ mice compared to WT (*P* < 0.05 and *P* < 0.0994, respectively), suggesting transitions from wake to anesthetization, or sleep, are impacted more than transitions from anesthetization/sleep to wake. On the acoustic startle test, Kir6.2^−/−^ mice trended toward a decreased startle response compared to controls (Fig. 3*H*, *P* = 0.0754), further suggesting subtle alterations in arousal. Changes in theta activity are not only linked with arousal, but also anxiety (41, 42). Therefore, we explored anxiety-like behaviors in Kir6.2^−/−^ mice through a walking initiation task and locomotor activity. Kir6.2^−/−^ mice are quicker to investigate a novel context compared to WT on a walking initiation task (Fig. 3*I*, *P* < 0.01), suggesting decreased anxiety-like behaviors. While no overt motor impairments were observed in Kir6.2^−/−^ mice during an open field test, we observed a trend where Kir6.2^−/−^ mice explored the center of the cage more than WT mice (Fig. 3*J*, *P* = 0.0698), which can suggest an anxiolytic phenotype. Taken together, decreased alpha and theta activity are associated with decreased arousal and anxiety-like behaviors in the Kir6.2^−/−^ mice.

Since Kir6.2-K_ATP_ channel deletion impacts theta activity, we also quantified the relative ratio of theta-to-gamma power, a measure of memory and cognitive control. In the Kir6.2^−/−^ mice, theta:gamma ratio was decreased across wake, NREM, and REM (Fig. 3*K*, *P* < 0.01, *P* < 0.0001), suggesting Kir6.2^−/−^ mice may suffer from memory impairment (43, 44). Therefore, we performed the MWM. While spatial memory was largely intact, we found a trend where Kir6.2^−/−^ mice spend less time in the target quadrant during the probe trial compared to WT (Fig. 3*L*, *P* < 0.0617), which suggests subtle changes in memory (45).

### Kir6.2-K_ATP_ Channel Deletion Delays Wake-to-Sleep Transitions, Reduces Sleep Time, and Shifts Relative Power Toward High Frequency Activity.

**Fig. 4.**
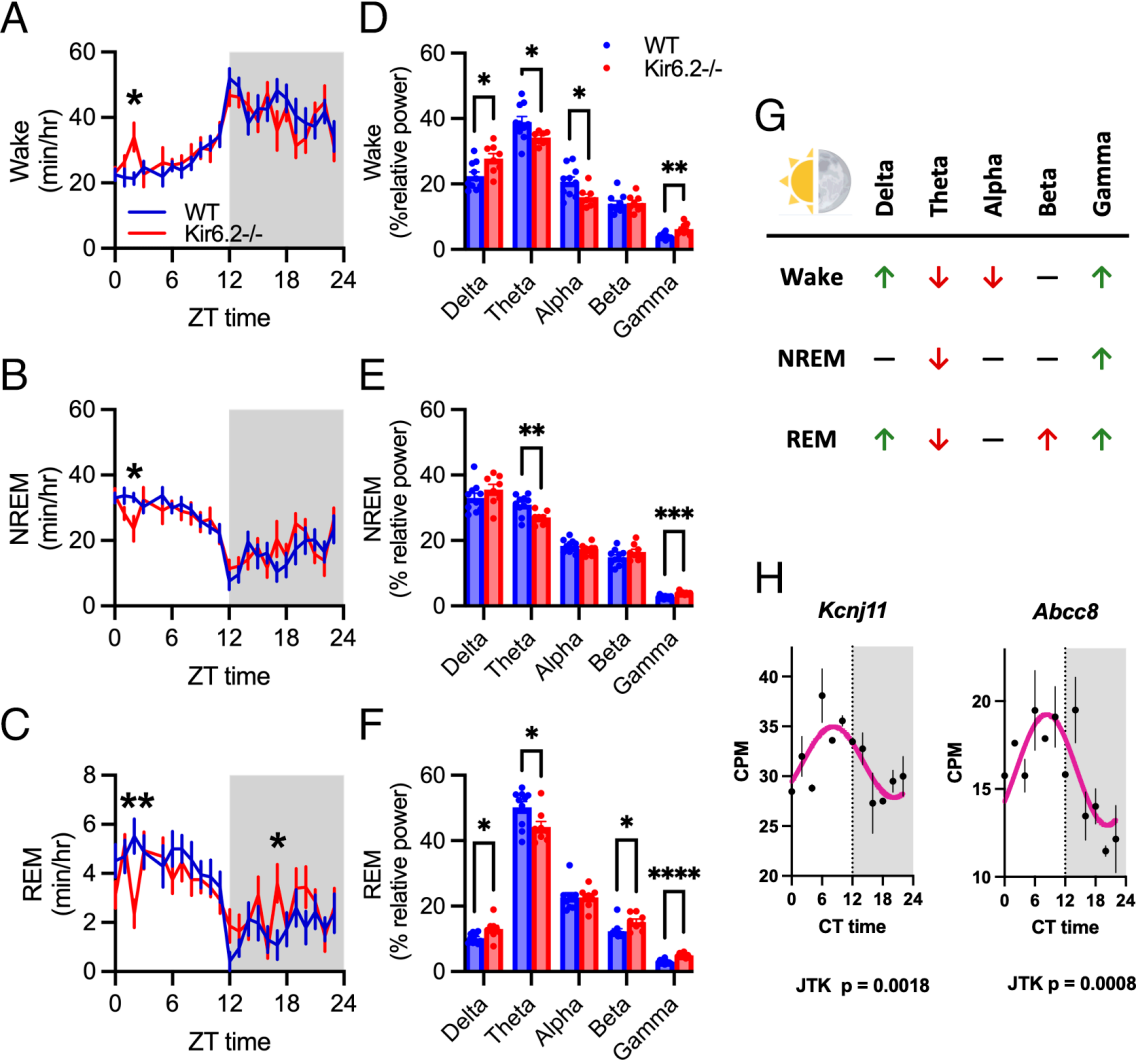
Kir6.2^−/−^ mice have reduced sleep time and a relative shift in EEG power from theta to gamma across sleep/wake states. (*A*–*C*) Kir6.2^−/−^ mice have increased wake, decreased NREM, and decreased REM sleep at ZT3 (*P* < 0.05, two-way ANOVA). Kir6.2^−/−^ mice also have increased REM at ZT17 (*P* < 0.05). (*D*) Kir6.2^−/−^ mice lose theta and alpha power and gain delta and gamma power during wake (*P* < 0.05, two-way ANOVA). (*E*) Kir6.2^−/−^ mice lose theta and gain gamma power during NREM (*P* < 0.01, *P* < 0.001, two-way ANOVA). (*F*) Kir6.2^−/−^ lose theta power and gain delta, beta, and gamma power during REM (*P* < 0.05, *P* < 0.05, *P* < 0.05, *P* < 0.0001, two-way ANOVA). (*G*) Summary table. (*H*) *Kcnj11* and *Abcc8* genes are rhythmic over 24-h day (*P* = 0.0018, *P* = 0.0008, RAIN). Data reported as means ± SEM. n = 8 to 11 mice/genotype for EEG analysis, n = 2 mice for gene rhythmicity.

Since alpha and theta activity are strongly associated with arousal and sleep pressure, we explored whether Kir6.2^−/−^ mice have impairments in sleep. Sleep staging was performed using EEG/EMG and average minutes per hour of wake, NREM, and REM were calculated for Kir6.2^−/−^ and WT mice. Over the 24 h day, Kir6.2^−/−^ mice show a trend toward more time awake during the light period compared to WT (ZT0-12; *SI Appendix*, Fig. S3*A*, *P* = 0.0709). This was driven by changes in the sleep/wake times at ZT0-3 when mice should transition to sleep (Fig. 4 *A*–*C*). At ZT3, wake time was increased (*P* < 0.05), suggesting a delayed transition to sleep in Kir6.2^−/−^ mice.

Next, we quantified the distribution of relative EEG power across sleep/wake states to assess the quality of sleep/wake states and the ability to transition between states (Fig. 4 *D*–*G*). During wake, there was increased relative delta and gamma power (Fig. 4*D*, *P* < 0.05, *P* < 0.01), with a corresponding decrease in theta and alpha power in Kir6.2^−/−^ mice (Fig. 4*D*, *P* < 0.05). Shifts toward increased delta activity and decreased theta power suggest increased homeostatic sleep drive but decreased sleep propensity (46–49). Wake also displayed increased relative gamma activity, suggesting heightened arousal and complex cognitive processing, which could explain a decreased likelihood to transition to sleep. It also explains why humans with mutations in *KCNJ11*, which results in Kir6.2-K_ATP_ channel dysfunction, suffer from profound seizure activity (50, 51). In NREM sleep, Kir6.2^−/−^ mice had decreased relative theta power with a shift toward increased gamma (Fig. 3*E*, *P* < 0.01, *P* < 0.001). Theta power should build over NREM bouts to drive REM sleep, suggesting an inability to switch between sleep/wake states. NREM sleep is traditionally composed of low frequency delta activity so increased high frequency gamma activity, known to drive depolarization or UP states (52), decreases NREM sleep quality. In REM, Kir6.2^−/−^ had increased relative delta, beta, and gamma power (Fig. 4*F*, *P* < 0.05, *P* < 0.0001), with a decrease in relative theta power (*P* < 0.05). This suggests that the integrity of REM sleep is also compromised since theta power should dominate REM sleep (53). Across all sleep/wake states, a shift from decreased theta and increased gamma power is observed, suggesting an uncoupling of theta:gamma EEG activity and tightly coordinated neuronal activity.

Given that changes in sleep/wake cycles occurred at specific times of day, we explored whether gene expression of Kir6.2-K_ATP_ channels was rhythmic. Kir6.2-K_ATP_ channels are composed of Kir6.2 (*Kcnj11)* subunits and Sur1 (*Abcc8)* sulfonylurea binding site. To assess gene rhythmicity, wildtype (WT) mice were killed every 2 h over a 24-h time period and Rhythmicity Analysis Incorporating Nonparametric methods (RAIN) analysis, a commonly used algorithm for identifying circadian oscillation in transcript expression in RNAseq data, was performed for *Kcnj11* and *Abcc8* (54). Both *Kcnj11* and *Abcc8* are rhythmic, with peak expression during the light period at ZT10-12 and decreased expression during the dark period (Fig. 4*H*). This suggests Kir6.2-K_ATP_ channel expression is under circadian control, which may provide one explanation for why the changes in excitability and sleep/wake states are more pronounced at specific times of day.

### Kir6.2-K_ATP_ Channel Deletion Causes Phase Shifts in Diurnal Rhythms of ISF Lactate, Which Corresponds with Delays in Sleep–Wake Transitions.

**Fig. 5.**
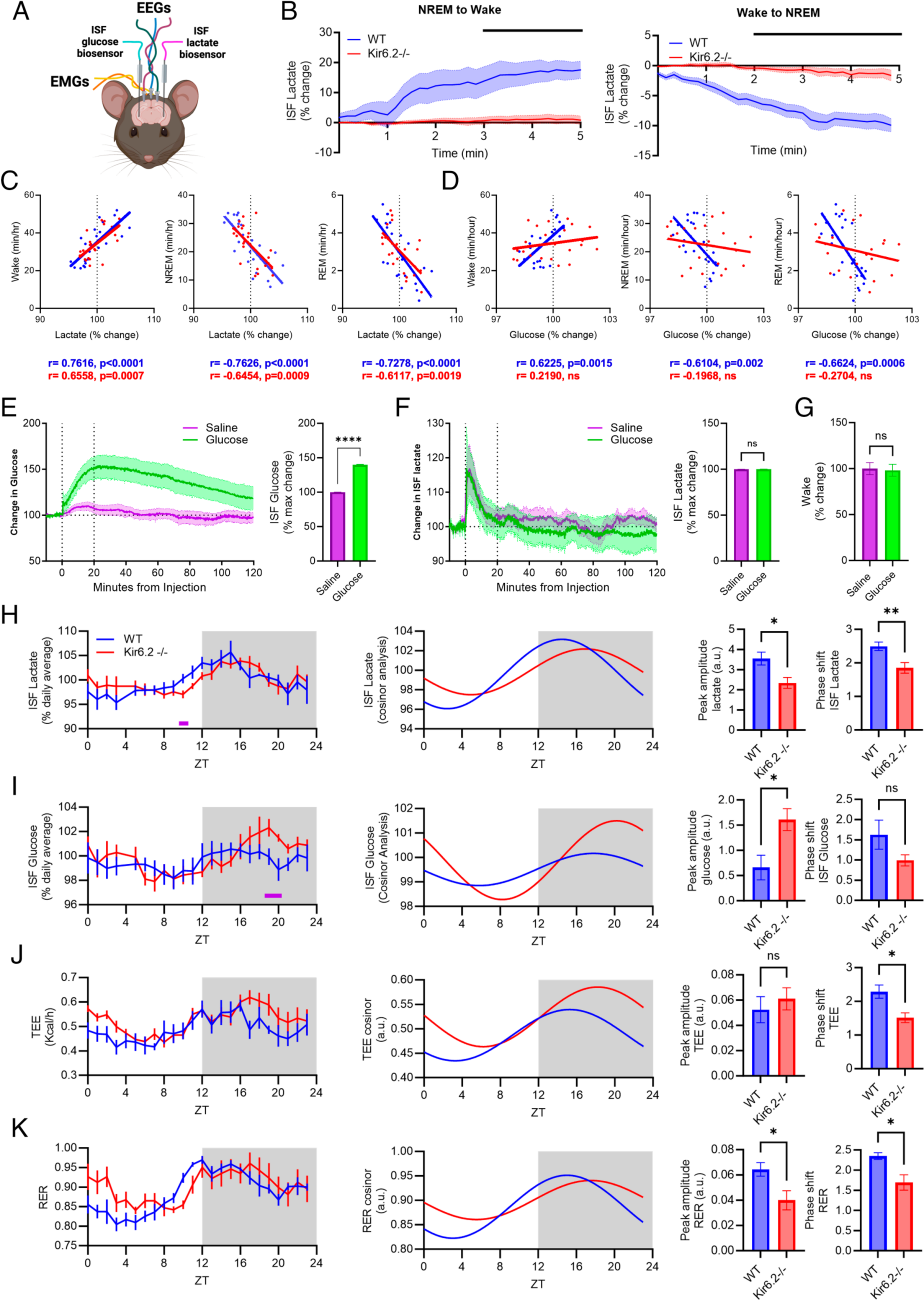
Peripheral metabolic rhythms mirror ISF lactate rhythms in Kir6.2^−/−^ mice. (*A*) Schematic of EEG/EMG/biosensor implants. (*B*) ISF lactate fluctuations are lost during NREM to wake and wake to NREM transitions in Kir6.2^−/−^ mice (*P* < 0.05, two-way ANOVA). (*C*) ISF lactate is positively correlated with increases in time spent in wake and negatively correlated with time spent in NREM and REM sleep in both Kir6.2^−/−^ and WT mice (Pearson’s R correlation). (*D*) ISF glucose is positively correlated with time spent in wake and negatively correlated with time spent in NREM and REM sleep in WT mice, but this relationship is lost in Kir6.2^−/−^ mice (Pearson’s R correlation). (*E*) Glucose challenge increases ISF glucose in Kir6.2^−/−^ mice (*P* < 0.0001, one-way ANOVA, unpaired *t* test). (*F*) Glucose challenge does not increase in ISF lactate in Kir6.2^−/−^ mice (one-way ANOVA, unpaired *t* test). (*G*) A glucose challenge does not increase time in wake in Kir6.2^−/−^ mice. (*H*) The rise in ISF lactate preceding the light–dark switch is delayed in the Kir6.2^−/−^ (*P* < 0.05, two-way ANOVA). ISF lactate rhythms show decreased amplitude and a phase shift in Kir6.2^−/−^ mice (*P* < 0.05, *P* < 0.001, unpaired *t* test). (*I*) The peak in ISF glucose levels is increased in Kir6.2^−/−^ mice at ZT19-20 compared to WT (*P* < 0.05, unpaired *t* test). (*J*) Total energy expenditure (TEE) rhythms are phase shifted in Kir6.2^−/−^ mice v WT (*P* < 0.05, unpaired *t* test). (*K*) Respiratory exchange ratio (RER) rhythms have decreased amplitude and a phase shift in Kir6.2^−/−^ mice (*P* < 0.05, unpaired *t* test). Data reported as means ± SEM. ISF lactate, ISF glucose, and sleep/wake transitions (n = 8 to 18 mice/genotype). Correlations n = 8 to 11 mice/genotype, TEE and RER n = 5 mice/genotype.

Since our EEG data suggest problems with state switching in Kir6.2^−/−^ mice, we analyzed whether there were alterations in ISF lactate, a metabolic biomarker of sleep/wake transitions, during NREM-to-wake and wake-to-NREM transitions (Fig. 5*A*) (3, 5, 55). We found that as Kir6.2^−/−^ mice transition from sleep to wake, ISF lactate does not increase as rapidly as it does in WT mice (Fig. 5*B*, *P* < 0.05). Similarly, ISF lactate levels do not drop as quickly during wake-to-NREM transitions in Kir6.2^−/−^ mice as they do in WT mice (Fig. 5*B*, *P* < 0.05). We then explored whether changes in ISF lactate levels correlated with time spent in sleep/wake states, where ISF lactate should ultimately rise with wake and fall with sleep. ISF lactate levels were positively correlated with wake in both Kir6.2^−/−^ and WT mice (Fig. 5*C*, *P* < 0.0001, *P* < 0.0007). In NREM and REM, the opposite pattern was observed where ISF lactate levels were negatively correlated with time spent in sleep in Kir6.2^−/−^ and WT mice (Fig. 5*C*, *P* < 0.0001, *P* < 0.0009, *P* < 0.0019). This suggests that while ISF lactate levels ultimately do fluctuate with sleep/wake states, ISF lactate dynamics are compromised in Kir6.2^−/−^ mice, reinforcing the idea that acute changes in ISF lactate are necessary for efficient transitions between sleep/wake states.

We also explored how fluctuations in ISF glucose correlated with sleep/wake states (Fig. 5*D*). While correlations between ISF glucose and sleep/wake states were present in WT mice and mirrored changes in ISF lactate, all correlations between ISF glucose and sleep/wake states were lost in Kir6.2^−/−^ mice (Fig. 5*D*). This suggested that ISF glucose and ISF lactate were uncoupled in Kir6.2^−/−^ mice. We previously demonstrated that a peripheral hyperglycemic challenge is sufficient to stimulate wake through ISF lactate production (7). Therefore, we explored how Kir6.2^−/−^ mice responded to a peripheral hyperglycemic challenge. ISF glucose increased in Kir6.2^−/−^ brains following a glucose challenge (Fig. 5*E*, *P* < 0.0001); however, neither ISF lactate levels nor time spent awake increased in Kir6.2^−/−^ mice (Fig. 5 *F* and *G*). This shows that Kir6.2-K_ATP_ channels are necessary to couple ISF glucose with ISF lactate to modulate sleep/wake transitions.

Next, we investigated how ISF levels of glucose and lactate fluctuated over the 24-h day since Kir6.2-K_ATP_ channel expression is rhythmic. Fluctuations in ISF lactate peak during the dark period in Kir6.2^−/−^ and WT mice (Fig. 5*H*), but ISF lactate has a lower peak amplitude and a phase shift in Kir6.2^−/−^ mice compared to WT (Fig. 5*H*, *P* < 0.05, *P* < 0.01). This suggests a delay to rise in ISF lactate at the light–dark transition. While ISF glucose levels also peak in the dark period, the peak at ZT19-20 is greater in Kir6.2^−/−^ mice compared to WT (Fig. 5*I*, *P* < 0.05). This suggests a shift in ISF lactate dynamics due to Kir6.2-K_ATP_ channel deficiency, especially at light–dark transitions.

Next, we explored whether alterations in metabolic rhythms were restricted to the brain in Kir6.2^−/−^ mice since the Kir6.2^−/−^ mice are a global knockout. Kir6.2^−/−^ and WT mice underwent indirect calorimetry to assess changes in whole body metabolism (56). TEE, a measure of an animal’s energy requirements, and RER, a metric of fuel preference, were largely unchanged in Kir6.2^−/−^ mice compared to WT (Fig. 5 *J* and *K*). We did find that fluctuations in ISF lactate were more strongly correlated with peripheral metabolism than ISF glucose (Fig. 5 *J* and *K* and *SI Appendix*, Fig. S4). While fluctuations in TEE and RER were rhythmic in both groups, both TEE and RER exhibited a similar phase delay as ISF lactate in the Kir6.2^−/−^ mice (Fig. 5 *J* and *K*, *P* < 0.05). TEE and RER were also strongly correlated with time spent in sleep/wake states (*SI Appendix*, Fig. S5) and ISF lactate (*SI Appendix*, Fig. S4), but the relationship between either TEE or RER and ISF glucose were lost in Kir6.2^−/−^ mice (*SI Appendix*, Fig. S4). Despite phase shifts and alterations in peak amplitudes, ISF lactate, ISF glucose, and peripheral metabolic measurements retained rhythmicity in Kir6.2^−/−^ mice (*SI Appendix*, Table S1). Overall, this illustrates that changes in brain and peripheral metabolism are tightly coupled, that changes in brain metabolism in the Kir6.2^−/−^ mice could be driven in part by changes in peripheral metabolism, and that TEE and RER correlate strongly with ISF lactate fluctuations and sleep/wake states.

## Discussion

Healthy brain function relies on the dynamic interplay between metabolism and excitability, where different sleep/wake states have their own metabolic- and activity-dependent profiles. The ability of the brain to execute complex behavior or conversely, engage in restorative states, relies on coordinated changes in metabolism and neuronal excitability. While it is well established that patterns of neuronal activity underlie changes in behavior, less is known about how metabolic flexibility and glycolytic flux can respond to and drive changes in arousal. When considering glucose metabolism as a contributor to sleep/wake states, our work demonstrates that glucose must be considered as both a fuel source for ATP generation, but also as a substrate for nonoxidative metabolism, biosynthesis, and redox regulation. Our data suggest alterations in glycolytic flux, not primary changes in mitochondrial function, have profound effects on cellular function, demonstrating its importance for the stability and flexibility of sleep/wake states.

^13^C-glucose SIRM experiments illustrated that Kir6.2-K_ATP_ channel deletion alters glycolytic flux where glucose is used for glycolysis at the expense of neurotransmitter synthesis. It is notable that the change in glycolytic flux did not occur as a result of an overall glucose shortage, but as a reaction to the way the brain, and most likely neurons, detect and use glucose as a substrate for biosynthesis or specific physiological processes. Previous studies demonstrated that loss of K_ATP_ channel activity reduces neuronal activity via reductions in the membrane potential, yet this study suggests that glycolytic flux may be driving some of these changes. Glycolysis is believed to be essential for maintaining resting membrane potentials at the synapse (57), which could explain why Kir6.2^−/−^ neurons prioritize it over mitochondrial respiration or biosynthesis. Regardless, this subtle alteration in glycolytic flux was sufficient to decrease cortical EEG power, the integrity of sleep/wake states, and the flexibility to transition between states. We found that absolute power was dampened across all sleep/wake states in Kir6.2^−/−^ mice, with a shift in relative power from lower to higher frequency activity. The loss of theta power is interesting since this was observed in wake, NREM, and REM. The likelihood of transitioning between sleep/wake states appears to be driven, in part, by theta power (58, 59). During wake, the ability to attend, perform, and sustain different levels of arousal depends upon theta activity in the anterior cortex (60). Therefore, this study identifies a role for Kir6.2-K_ATP_ channels in regulating behaviors associated with theta power. Furthermore, alpha power is associated with drowsiness or quiet wakefulness, which is necessary for the transition to sleep. The reduction of alpha power during wake reinforces the idea that Kir6.2-K_ATP_ channels are important for modulating arousal and state-shifting. Our conclusions are further supported by clinical studies where individuals with *KCNJ11* mutations report higher incidences of problems with sleep, anxiety, and attention (22).

The behavioral manifestations of Kir6.2-K_ATP_ channel deficiency were nuanced. Ultimately, whether looking at arousal, anxiety, cognition, or sleep, mice lacking K_ATP_ channel activity largely retained the ability to complete tasks or maintain sleep/wake homeostasis. However, the robustness of a specific behavior or the ease to switch between behavioral states was compromised in Kir6.2^−/−^ mice. This suggests that Kir6.2-K_ATP_ channels regulate metabolic flexibility and arousal. Again, this highlights the idea that metabolic flexibility is as important as total energy availability when considering changes in physiology and behavior.

Another key observation is that Kir6.2-K_ATP_ channels are necessary for glucose-lactate coupling, which is integral for sleep/wake transitions. ISF lactate levels are a biomarker for sleep. Increased lactate triggers wake, while ISF lactate levels drop for the transition to sleep. Our ^13^C-glucose experiments show that: 1) the relative production of lactate in the Kir6.2^−/−^ brain is higher than WT, 2) the total abundance of brain lactate is comparable between Kir6.2^−/−^ mice and WT, and 3) yet the pool of ISF lactate is reduced or less dynamic in Kir6.2^−/−^ mice. This demonstrates that Kir6.2-K_ATP_ channels not only act as metabolic sensors to couple metabolism with excitability, but also regulate the extracellular flux of lactate which has pleiotropic effects on cortical activity, sleep, and behavior. Herein, we demonstrate that alterations in lactate dynamics due to Kir6.2-K_ATP_ channel deficiency underlies the integrity of and transition between sleep/wake states. Phase delays in ISF lactate accompany disruptions in sleep homeostasis, most notably in wake-to-sleep transitions. While it is known that K_ATP_ channels are localized to the hypothalamus and play a role in orexinergic neuronal functioning, we show that *Kcnj11* and *Abcc8*, the genes regulating Kir6.2^−/−^ and Sur1 expression, are rhythmic. This shows an interesting interplay between sleep/wake cycles and circadian rhythms, where K_ATP_ channels may serve as a metabolic zeitgeber for both processes.

Some limitations of this study exist. We used a whole-body knockout of Kir6.2-K_ATP_ activity, which means that alterations in brain metabolism and excitability could be driven, in part, by changes in peripheral metabolism. While Kir6.2-K_ATP_ channels are located on other cell types such as cardiac and skeletal muscle, >98% of Kir6.2-K_ATP_ channels in the brain are located on neurons, and more specifically, glutamatergic, excitatory neurons. Given the alterations in glucose utilization, reduction in neurotransmitter synthesis, and dampened cortical EEG activity, we suggest that this is due to the loss of neuronal Kir6.2-K_ATP_ channel more so than other cell types (e.g., OPCs, microglia, astrocytes, vascular cells). Our data are consistent with recent studies exploring the physiological consequences of human mutations in the *KCNJ11* gene that result in clinical developmental delay, epilepsy, and neonatal diabetes or DEND syndrome (50, 51). Here, *KCNJ11* mutations also altered neuronal excitability, seizure susceptibility, and gamma activity. Our data link these changes in excitability with changes in glycolytic flux and explore their role in sleep.

Taken together, our studies describe how Kir6.2-K_ATP_ channels play an important role in glycolytic flux, where glucose serves as both a metabolite and biosynthetic substrate for the brain. Kir6.2-K_ATP_ channel deficiency causes subtle changes in glycolytic flux that are associated with alterations in cortical EEG activity, leading to deficits in arousal, attention, and sleep/wake architecture. This highlights the important relationship between metabolic flexibility and the maintenance of different sleep/wake states. Ultimately, this has implications for many diseases including, type-2-diabetes, DEND syndrome (50, 51), and Alzheimer’s disease (10, 22), where alterations in K_ATP_ channels are known to play a role in disease pathogenesis.

## Materials and Methods

### Mice.

Male and female Kir6.2-deficient (Kir6.2^−/−^) and WT (WT; both B6C3F1/J mixed genetic background) were used in all experiments. Mice were group housed, given food and water ad libitum, and maintained on a 12:12 light/dark cycle. All procedures were carried out in accordance with an approved IACUC protocol from either Washington University in St. Louis, Wake Forest School of Medicine, or the University of Kentucky College of Medicine.

### *Kcnj11* and *Abcc8* Expression in the Mouse Brain.

To determine the cell type distribution of K_ATP_ channel expression in the mouse brain, we analyzed gene expression of *Kcnj11* and *Abcc8*, which code for Kir6.2 and Sur1, respectively, in two publicly available datasets (brainRNAseq.org and portal.brain-map.org/atlases-and-data/bkp/abc-atlas) (23, 24). Using the brainRNAseq.com database, we determined the expression level of *Kcnj11* and *Abcc8* in neurons, oligodendrocytes precursor cells (OPCs), oligodendrocytes, astrocytes, microglia/macrophages, endothelial cells as previously described (23, 24). Using the portal.brain-map.org/atlases-and-data/bkp/abc-atlas from the Allen Brain Institute (24), we further explored the expression of *Kcnj11* and *Abcc8* on different subtypes of neurons, including glutamatergic, GABAergic, dopaminergic, or serotonergic neurons, as well as other cell types, including astrocytes, ependymal cells, OPCs, oligodendrocytes, immune cells, or pineal cells as previously described (23, 24). Data were pulled using Python from the Allen Brain Cell Atlas, which is hosted on Amazon Web Services (AWS) in an S3 bucket. The full 4 million cell 10X dataset was used, which is from 3 different experiments (10Xv2, 10Xv3, and 10XMulti). The anndata package was used to load the log2 normalization files (expression data) and pandas for the data manipulation. For the heatmap, data were pseudobulked by taking the average (or the z score) by cell type and used matplotlib for the plotting/visualization.

### Synaptic and Nonsynaptic Mitochondrial Isolation.

The mitochondrial isolation protocol was adapted from previously established methods (61, 62). All steps were performed at 4 °C. Briefly, 3- to 4-mo-old Kir6.2^−/−^ and WT male mice (n = 6 mice/genotype) were euthanized by cervical dislocation at ~9 am (ZT3), and the brain cortices were homogenized in a Teflon-glass Dounce homogenizer containing mitochondrial isolation buffer (MIB) (215 mM mannitol, 75 mM sucrose, 0.1% BSA, 20 mM HEPES, 1 mM EGTA, pH adjusted to 7.2 with KOH). The homogenates were transferred to 2 mL microcentrifuge tubes and centrifuged at 1,300×*g* for 3 min. The supernatants were transferred to fresh tubes and centrifuged at 13,000×*g* for 10 min. The resulting crude mitochondrial pellet was resuspended in 450 µL of MIB. A double-layer Ficoll gradient was prepared with 2 mL of 7.5% Ficoll layered over 2 mL of 10% Ficoll. The mitochondrial suspension was layered onto the gradient and centrifuged at 100,000×*g* for 30 min in an ultracentrifuge (Beckman Coulter Optima-XPN). The intermediate layer containing neuro-synaptosomes was collected into a separate 2 mL tube, while the pellet containing nonsynaptic mitochondria was collected into a 1.5 mL tube in MIB. The neurosynaptosomal fraction was diluted with MIB, pelleted, and resuspended in 450 µL of isolation buffer. Synaptic mitochondria were released using a pressurized nitrogen cell disruptor at 1,200 psi for 10 min at 4 °C and further purified using the Ficoll gradient as described. Both synaptic and nonsynaptic mitochondrial pellets from the ultracentrifugation steps were washed in 1.5 mL of MIB and pelleted at 13,000×*g* for 10 min at 4 °C. The final mitochondrial pellets were resuspended in 100 µL of MIB. Protein concentration was determined using the bicinchoninic acid (BCA) protein assay kit (Pierce, Cat #23227).

### Mitochondrial Bioenergetics.

Mitochondrial bioenergetics were measured using the Seahorse XFe96 Extracellular Flux Analyzer (Agilent Technologies, USA) as per the previously established protocol (63). Synaptic and nonsynaptic mitochondria were diluted in mitochondrial respiration buffer (125 mM KCl, 0.1% BSA, 20 mM HEPES, 2 mM MgCl_2_ and 2.5 mM KH_2_PO_2_ adjusted to pH 7.2 with KOH). A total of 1 µg of nonsynaptic mitochondria and 2 µg of synaptic mitochondria were loaded per well. OCR were assessed under various respiratory states using substrates, inhibitors, and uncouplers of the electron transport chain, as per a lab-optimized protocol. Sensor cartridges were hydrated overnight at 37 °C. Injection ports A–D were loaded with diluted combinations to achieve final concentrations of 5 mM pyruvate, 2.5 mM malate, and 1 mM ADP (Port A); 1 µM oligomycin A (Port B); 4 µM FCCP (Port C); and 0.1 µM rotenone with 10 mM succinate (Port D). Purified mitochondria (1 µg/well for nonsynaptic and 2 µg/well for synaptic) were adhered to XFe96 assay plates via centrifugation (3,900×*g* for 10 min at 4 °C), overlaid with 145 µL prewarmed to 37 °C respiration buffer, and analyzed after calibration. Data were collected through sequential injections, mixing, equilibration, and OCR measurements. Outputs included State III respiration (pyruvate, malate, ADP), State IV respiration (oligomycin), uncoupled respiration complex-I (FCCP), and uncoupled respiration complex-II (rotenone/succinate).

### ^13^C-Glucose Oral Gavage and Tissue Collection for Stable Isotope Resolved Metabolomics.

Kir6.2^−/−^ and WT mice were single housed and fasted for 4 h starting ~9 am clock time (ZT3). Mice were given an oral gavage of 250 μL of 32 mg/ml ^13^C-glucose (Cambridge Isotope Laboratories Inc., #110187-42-3) an estimated dose of 2 g/kg per animal for mice starting ~1 pm clock time (ZT7). 45 min post gavage, mice were cervically dislocated, and brain tissue was rapidly extracted. Samples were washed and flash frozen in liquid nitrogen. Mice were killed between 2 pm and 3 pm clock time (ZT8-9). Stable isotope resolved metabolomics was performed as previously described (25).

### Electroencephalography (EEG)/Electromyography (EMG) Surgery.

Three- to 6-mo-old Kir6.2^−/−^ and WT mice (N = 8 to 11 mice/genotype) were anesthetized with isoflurane and underwent stereotaxic surgery. Biosensor guide cannulas (MD-2255, BASi Research Products) were implanted bilaterally into the hippocampus (A/P −3 mm, M/L ± 3 mm, D/V −1.8 mm). Two stainless steel skull screws (MD-1310, BASi Research Products) were placed into the skull over the right frontal cortex (A/P 1 mm, M/L −1 mm) and left parietal cortex (A/P −2 mm, M/L −1 mm). These sites served as recording electrodes. A third reference electrode was placed over the cerebellum (A/P −6 mm, M/L 0 mm). Insulated wire leads soldered to an EEG/EMG/Biosensor headmount (8,402, Pinnacle Technology) and attached to the implanted skull screws for EEG recordings. A set of stainless steel wires were implanted into the nuchal muscles for EMG recordings. The screws, biosensor cannulas, EEG lead wires, and headmount were secured to the skull using acrylic dental cement (Reliance DuraLay). Postsurgery, mice were single housed and allowed to recover in recording cages (8,288, Pinnacle Technology) placed into sound attenuating chambers (ENV-017 M Med Associates). Mice were maintained on 12:12 light/dark cycle.

### Biosensor Calibration.

Biosensors were calibrated before insertion in mice per the manufacturer’s protocol (Pinnacle Technologies) and as described elsewhere (5). Briefly, using the manufacturer's calibration stage (7,052, Pinnacle Technology), biosensors were inserted into 1xPBS at 37 °C and allowed to stabilize for a baseline measurement. Two consecutive injections of the analyte of interest (L-Lactate or D-Glucose) were injected into this heated 1xPBS solution. A third interference analyte injection (ascorbic acid) was conducted. Finally, two additional consecutive injections of the analyte of interest were conducted. Biosensor readings were allowed to stabilize in between all injections. The resulting readout of all biosensors used in this experiment was strong, stepwise response to analytes of interest, and no response to the interferant analyte. The average change in response to each analyte injection was converted into a constant for that individual biosensor to be used for analyzing experimental data.

### EEG/EMG, Glucose Biosensor, and Lactate Biosensor Recording.

72 h postsurgery, mice were briefly anesthetized with isoflurane and two amperometric biosensors specific for either glucose or lactate (7,004, Pinnacle Technology) were inserted into the guide cannula (n = 8 to 11 mice/genotype) into the left and right hippocampus. Biosensors were connected to a preamplifier [100x amplification, EEG high pass filter: 0.5 Hz, EMG high pass filter: 10 Hz (8406-5SL, Pinnacle technology)] and inserted into the mouse’s headmount. The preamplifier was connected to the data acquisition system via a commutator (8,401, Pinnacle Technology). Data sampling was transmitted at 250 Hz for EEG/EMG and 1 Hz for the biosensors. Mice were unanesthetized and freely allowed to move about their cages for the duration of recordings. Recordings began immediately. Analysis was only performed after a stable baseline was reached for EEG/EMG and biosensors (Sirenia Acquisition software). 24-h recordings of EEG/EMG and biosensor data taken from day 2 of the recording were used to establish a diurnal rhythm (n = 8 to 11 mice per genotype).

### Biosensor Analysis.

24 h of biosensor data were analyzed from day 2 of recording. Nano-amp (nA) values per second were binned into 10 s averages. Using the calibration value for each biosensor, nA responses were converted into a concentration value of nanomoles (nM) for each analyte of interest. The average concentration value over the 24 h day was taken and used to create a percent of average concentration value for each 10 s bin. These 10 s percent of baseline values were then binned into 1 h bins. Biosensors that exceeded a max change of 10% from baseline average across 24 h were detrended using the detrend function in matlab to normalize data.

### Wake, NREM, and REM Scoring.

EEG/EMG data were exported and scored in 10 s epochs by a human trained in sleep scoring according to standard classifications (64) for wake (high frequency, low amplitude EEG, high amplitude EMG), NREM (low frequency, high amplitude EEG, low amplitude EMG), and REM (High frequency, low amplitude EEG, very low amplitude EMG) using Sirenia Sleep Software (n = 8 to 11 mice per genotype) for 24 h over the circadian day, beginning at lights on (ZT0).

### EEG Power Spectral Analysis.

EEG power spectral analysis was calculated using Sirenia Sleep Pro Software (Pinnacle Technology). A Fast Fourier transform (Hann window) was used to determine power spectra values for all epochs without artifacts and exported according to sleep and wake state. To determine absolute power, power spectra were binned in 1 Hz bins and graphed for wake, NREM, and REM. Relative power was generated by separating data into frequency bins in interest for sleep and wake behaviors for each 10 s epoch (delta 0.5 to 4 Hz, theta 4 to 8 Hz, alpha 8 to 13 Hz, beta 13 to 30 Hz, gamma 30 to 50 Hz). A percentage of total power value was calculated for each frequency band and graphed according to sleep–wake state.

### Sleep Transition Analysis.

Sleep–wake transitions were evaluated for 2 h post saline injection and, as, the EEG/EMG was dominated by wake and NREM bouts, REM epochs were excluded for this analysis (n = 8 to 18 mice/genotype). Inclusion criteria for which sleep and wake transitions to include were as follows: NREM bouts lasting ≥ 5 min and Wake bouts lasting ≥ 10 min. These criteria were used to capture a transition that resulted in either sustained wake or NREM sleep, since mice do not have consolidated sleep. The 60 s preceding a transition were used as a baseline of ISF lactate levels. This baseline was used to calculate a percent change in ISF lactate in the 5 min following a NREM to wake or wake to NREM transition.

### Saline and Glucose Injections with Biosensor and Sleep Analysis Protocol.

Intraperitoneal (IP) injections were given to Kir6.2^−/−^ mice only (n = 8) over a 2-d period: saline (0.9% NaCl, 2 g/kg) and glucose (50% dextrose, 2 g/kg). Injections were given across the light period at specific times [Day 1: 10 am (ZT4) and 3 pm (ZT9), Day 2: 10 am (ZT4)] and the order of injections was randomized to avoid any circadian effect. Biosensor data were exported and converted into mM data in 10 s bins as described above. Baseline biosensor data were defined as data 10 min before each injection. Postinjection biosensor bins were compared to the 10 min baseline and converted into a percentage change from baseline. % max change was determined by comparing % change from baseline of glucose injections relative to % change of baseline of saline injections. Changes in time spent in wake following the glucose injections were determined by comparing baseline % time in wake to % time in wake following each injection.

### Indirect Calorimetry.

Mice were transferred to the room containing Promethion Core metabolic system (Sable Systems International) and acclimated to room and single housing for 3 d while maintained on a 12:12 L:D cycle (56). Mice were then transferred into Promethion system for peripheral metabolic phenotyping (n = 5/genotype) for 4 d. Day 2 of indirect calorimetry data was used for all analysis.

### Arousal Challenge.

3 mo old WT and Kir6.2^−/−^ mice (n = 3 to 4/group) were given an intraperitoneal injection of ketamine/xylazine (dose = 108.6 mg/kg of ketamine, 16.8 mg/kg of xylazine). Two independent investigators observed mice for a loss of righting relax. “Induction time” is the duration it took for the mice to lose their righting relax postinjection. “Emergence time” was recorded as the duration it took for a mouse to display the first signs of consciousness, including motor movement and a righting reflex, since losing their righting relax.

### Behavioral Testing.

9-mo-old female Kir6.2^−/−^ and WT mice (N = 10 to 20 mice/genotype) were tested for behavioral differences in the Washington University Animal Behavior Core. Following 1 wk habituation and handling, mice were evaluated for differences in locomotor activity and exploratory behavior, walking initiation, MWM, and acoustic startle response. All tests were conducted during the light phase of the light/dark cycle, by an experimenter blind to the genotype of the mice.

### One-Hour Locomotor Activity and Exploratory Behavior.

To assess general activity levels and alterations in emotionality, mice were evaluated over a 1-h period in transparent (47.6 × 25.4 × 20.6 cm high) polystyrene enclosures. Each cage was surrounded by a frame containing a 4 × 8 matrix of photocell pairs, the output of which was fed to an on-line computer (Hamilton-Kinder, LLC, Poway, CA). The system software (Hamilton-Kinder, LLC) was used to define a 33 × 11 cm central zone and a peripheral or surrounding zone that was 5.5 cm wide with the sides of the cage being the outermost boundary. This peripheral area extended along the entire perimeter of the cage. Variables analyzed included the total number of ambulations and rearing on hindlimbs, as well as the number of entries, the time spent, and the distance traveled in the center area as well as the periphery surrounding the center.

### Walking Initiation.

In the walking initiation test, mice were placed on a flat surface in the center of a 21 × 21 cm square. The amount of time the mouse took to leave the square was recorded as a measure of initiation of movement.

### Morris Water Maze.

Morris Water Maze (MWM) testing was conducted as previously described (Yuede et al., 2021). Briefly, cued, place and probe trials were conducted in a galvanized steel pool, measuring 120 cm in diameter, and filled with opaque water (diluted nontoxic white tempera paint). The PVC escape platform measured 11.5 cm in diameter. A digital video camera connected to a PC computer with the computer software program ANY-maze (Stoelting Co.) tracked swimming pathway of the mouse to the escape platform and quantified path length, latency to find escape platform, and swimming speeds. On two consecutive days, animals received four cued trials to habituate to the swimming task procedure and control for any differences in swimming, visual, or motivational performance in the test. A red tennis ball atop a rod was attached to the escape platform and served as a visual cue for the platform. To prevent spatial learning, the escape platform was moved to a different quadrant location for each trial. The mouse was released from the quadrant opposite to the platform location and allowed 60 s to locate the platform. Once the mouse found the platform, it was allowed to remain there for 10 s before being returned to its home cage. 3 d following visible platform testing, the cue was removed from the platform, and it was submerged 1 cm under the water in a fixed location for the hidden platform tests to evaluate spatial learning. Animals received two blocks of two consecutive trials on five consecutive days, with an intertrial interval between 30 to 90 s and approximately 2 h separating trial blocks. The escape platform remained in the same quadrant location for all trials and distal cues were placed on the walls of the room to support spatial learning. The mouse was released from a different location for each trial on each day. The mouse was allowed 60 s to find the escape platform and allowed to sit on it for 10 s before being returned to its home cage. Cued and hidden platform trials were combined into blocks of two or four trials for analyses, respectively. 1 h following completion of hidden platform trials on the 5th d of training, the escape platform was removed from the pool and one 60 s probe trial was conducted to assess memory retention for the location of the platform.

### Acoustic Startle Response/Prepulse Inhibition.

Startle response to a 120 dB auditory stimulus pulse (40 ms broadband burst) and PPI (response to a prepulse plus the startle pulse) were measured concurrently in mice using the Kinder Scientific Startle Reflex system (Poway, CA). Beginning at stimulus onset, 1 ms force readings were averaged to obtain an animal's startle amplitude. A total of 20 startle trials were presented over a 20 min test period during which the first 5 min served as an acclimation period when no stimuli above the 70 dB white noise background were presented. The session began and ended by presenting 5 consecutive startle (120 db pulse alone) trials unaccompanied by other trial types. The middle 10 startle trials were interspersed with PPI trials (consisting of an additional 30 presentations of 120 dB startle stimuli preceded by prepulse stimuli of either 4, 12, or 20 dB above background (10 trials for each PPI trial type). A percent PPI score for each trial was calculated using the following equation: %PPI = 100*(ASRstartle pulse alone - ASRprepulse + startle pulse)/ASRstartle pulse alone.

### Jonckheere–Terpstra–Kendall (JTK) and RAIN Methods Analyses.

JTK analysis was performed using Rstudio package MetaCycle (65). Briefly, mean values for each time point were analyzed using MetaCycle and *P*-values, period, lag, and amplitude were reported. RAIN analysis was performed as previously described (54). Briefly, WT mice (n = 2) were euthanized every 2 h over the 24 h day, RNA isolated, and sequenced. Batch-corrected CPMs were fed into R package rain (version 1.32) to determine circadian gene expression in each dataset. Genes were considered rhythmic if the FDR-adjusted *P* value from RAIN was <0.01.

### Quantification and Statistical Analysis.

All EEG and sleep data were scored using the Sirenia Sleep Pro (Pinnacle Technologies) and exported for analysis. All of the statistical analysis was done using GraphPad Prism 10 (GraphPad Software, LLC). All data are reported as means with ± SEM. All details of statistical tests and corrections, subject numbers, and *P*-values are located in the figure legends. A *P*-value of *P* < 0.05 was used as a cutoff for determining statistical significance. For all groups, a Grubbs test was used to determine and remove outliers (alpha = 0.05). Specific statistical tests used were as follows:

Fig. 1: One-way ANOVA with Dunnett multiple comparisons correction.
Fig. 2: Two -way ANOVA with Tukey multiple comparisons correction; unpaired *t* test.
Fig. 3: Two-way ANOVA with Sidak or Tukey multiple comparisons correction; unpaired *t* test.
Fig. 4: Two-way ANOVA with Sidak or Tukey multiple comparisons correction for post hoc analysis; Jonckheere–Terpstra–Kendall analysis.
Fig. 5: Two-way ANOVA with Sidak or Tukey multiple comparisons correction for post hoc analysis; One-way ANOVA with Dunnett multiple comparisons correction for post hoc analysis; unpaired *t* test.
*SI Appendix*, Table S1: Jonckheere-Terpstra-Kendall analysis.
*SI Appendix*, Figs. S2 and S3: Unpaired *t* test.
*SI Appendix*, Figs. S4 and S5: Pearson’s R correlation.

## Supplementary Material

Appendix 01 (PDF)

Dataset S01 (XLSX)

## Data Availability

All study data are included in the article and/or supporting information.
