# Supplementary material for: ATP-sensitive potassium channels alter glycolytic flux to modulate cortical activity and sleep
Source: Proc Natl Acad Sci U S A. 2025 Feb 18;122(8):e2416578122. doi: 10.1073/pnas.2416578122 (PMC11874466; doi:10.1073/pnas.2416578122)
Supplement: Supplementary file 1 — Appendix 01 (PDF) [file pnas.2416578122.sapp.pdf]

Supporting Information for

ATP-sensitive potassium channels alter glycolytic flux to modulate cortical activity and sleep

Nicholas J. Constantino[1,2,3], Caitlin M. Carroll[5], Holden C. Williams[1,3], Hemendra J Vekaria[2,4], Carla M. Yuede[6,7], Kai Saito[2,3], Patrick W. Sheehan[7], J. Andy Snipes[1,3], Marcus E. Raichle [7,8,9,10,11*], Erik S. Musiek[7], Patrick G. Sullivan[2,4], Josh M. Morganti[2,3], Lance A. Johnson[1,3], & Shannon L. Macauley[1,2,3*]

Department of Physiology[1], Neuroscience[2], Sanders Brown Center on Aging[3], Spinal Cord and Brain Injury Research Center[4], University of Kentucky, Lexington, KY; Department of Psychiatry[5], Wake Forest School of Medicine, Winston-Salem, North Carolina; Department of Psychiatry[6], Neurology[7], Radiology[8], Neuroscience[9], Psychology & Brain Sciences[10], Biomedical Engineering[11], Washington University School of Medicine, St Louis, MO

*Marcus E. Raichle, Professor, Department of Radiology, Washington University School of Medicine, 660 S. Euclid Ave, St Louis, MO 63110

*Shannon L. Macauley, Associate Professor, Department of Physiology, University of Kentucky, 760 Press Avenue, Lexington, KY, 40508
Email: shannon.macauley@uky.edu.

Author Contributions: SLM and NJC conceived of the study. SLM, NJC, CMC, ESM, CMY, PGS, LAJ, JMM, and MER contributed to study design. NJC, CMC, HCW, CMY, HJV, PWS, and JAS performed experiments. SLM, NJC, CMC, HCW, CMY, HJV, KS, PWS, LAJ, and MER performed data analysis and data interpretation. NJC and SLM wrote the manuscript. All authors discussed the results and commented on the manuscript.

Competing Interest Statement: The authors declare no competing interests related to this publication.

Classification: Biological sciences; Neuroscience, Physiology

Keywords: K_{ATP} channels, metabolism, excitability, sleep, arousal

This PDF file includes:

- Supporting text
- Figures S1 to S5
- Tables S1

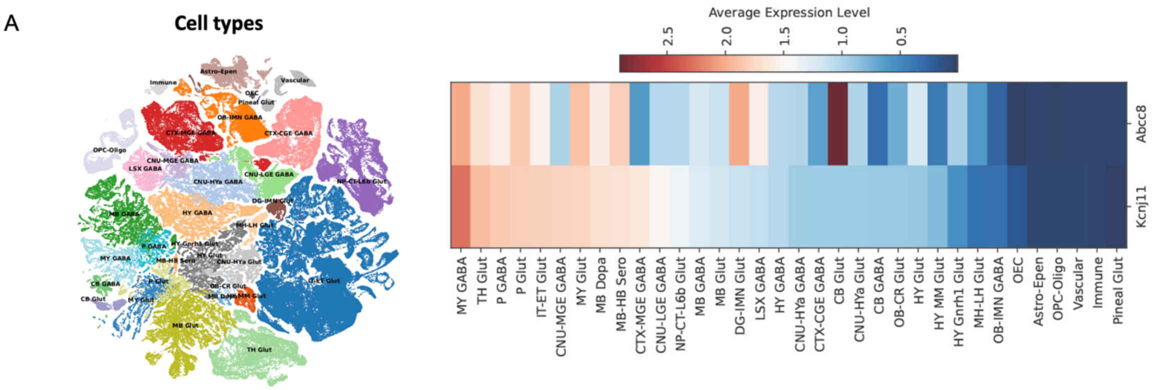

Figure S1. Average cell type expression of K_{ATP} subunits, *Kcnj11* and *Abcc8,* in the mouse brain. (A) Cell type UMAP plot and average transcript expression level (portal.brain-map.org/atlases-and-data/bkp/abc-atlas).

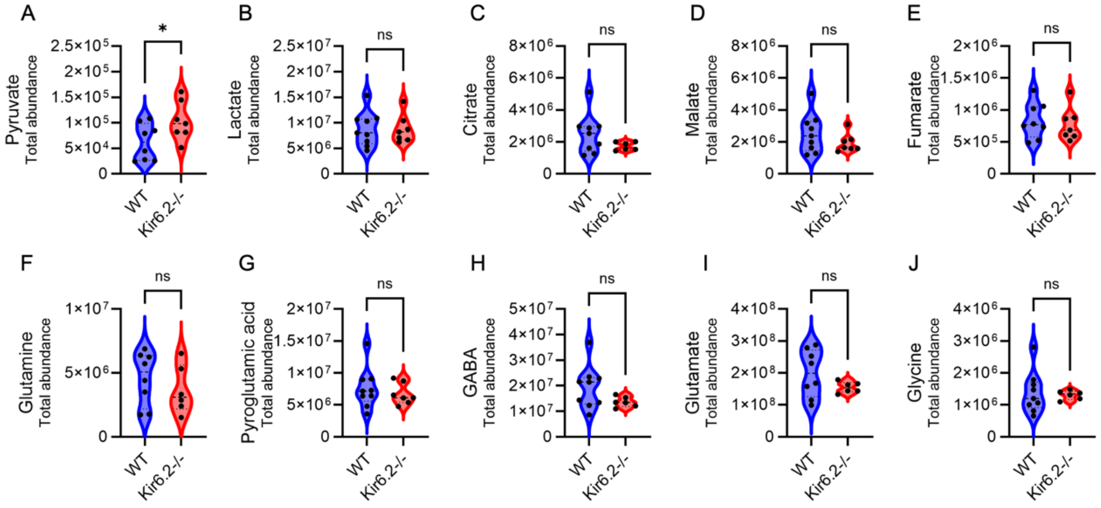

Figure S2. Brain metabolite total abundance quantified by stable isotope resolved metabolomics. (A-J) Total abundance is unaltered across groups. Data reported as means ± SEM. n = 8-9 mice/genotype. Significance determined via unpaired t-test.

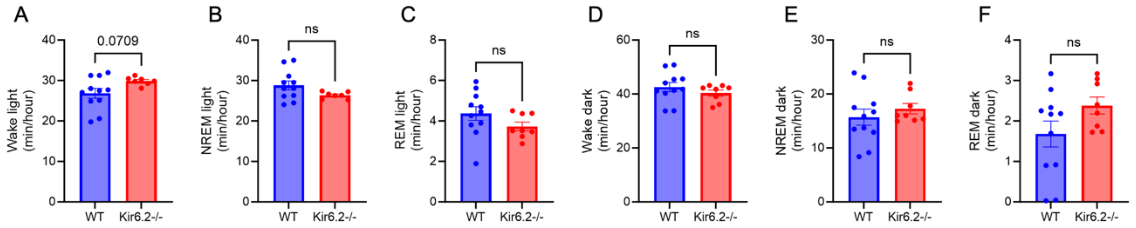

Figure S3. Time spent in sleep/wake states across light and dark periods. (A) Kir6.2-/- mice trend towards increased time spent in wake during the light period (p=0.0709). (B-C) There is no change in time spent in NREM and REM sleep in Kir6.2-/- mice during the light period. (D-F) No change in time spent in wake, NREM, or REM sleep in Kir6.2-/- mice during the dark period. Data reported as means ± SEM. n = 9-10 mice/genotype. Minute/hour sleep/wake state significance determined by unpaired t-tests.

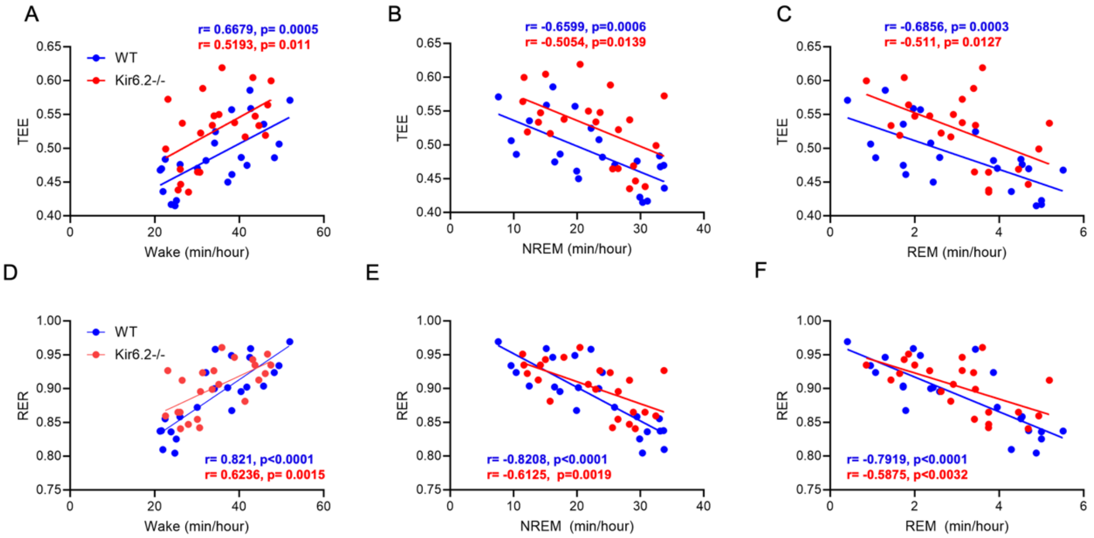

Figure S4. TEE and RER are correlated with sleep and wake states. (A-C) TEE is positively correlated with time spent in wake in both Kir6.2-/- and WT mice, and negatively correlated with NREM and REM sleep. (D-E) RER is positively correlated with time spent in wake in both Kir6.2-/- and WT mice, and negatively correlated with NREM and REM sleep. Data reported as means ± SEM. n = 5-11 mice/genotype. Significance determined by Pearson's R correlation.

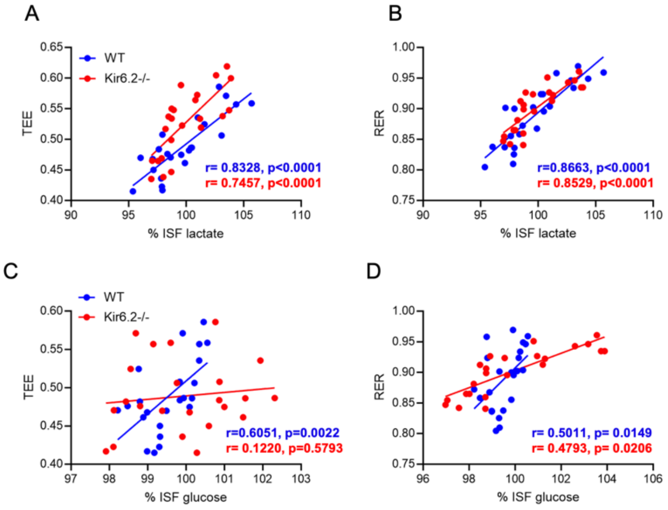

Figure S5. The relationship of peripheral and central metabolism. (A-B) TEE and RER are positively correlated with brain ISF lactate in both Kir6.2-/- and WT mice. (C) Kir6.2 -/- mice lose relationship between TEE and brain ISF glucose seen in WT mice. (D) Both Kir6.2-/- and WT mice RER are positively correlated with brain ISF glucose. Data reported as means ± SEM. n = 5-11 mice/genotype. Significance determined by Pearson's R correlation.

Table S1. Rhythmic analysis of peripheral and central metabolism. BH.Q = Benjamini-Hochberg procedure. ADJ.P = adjusted P value. Period = length of cycle. LAG = time of peak expression. Amplitude = peak expression. JTK analysis was performed for each genotype and condition.

	BH.Q	ADJ.P	Period	Lag	Amplitude
WT ISF Lactate	1.11E-08	1.39E-09	24	15	2.89325
Kir6.2-/- ISF Lactate	9.40E-04	8.30E-04	20	16.5	2.75599
WT ISF Glucose	1.12E-02	1.12E-02	20	16.5	0.84104
Kir6.2-/- ISF Glucose	1.00E-05	1.00E-05	23	19.5	1.38837
WT TEE	1.60E-04	1.20E-04	20	15	0.05657
Kir6.2-/- TEE	1.00E-05	1.00E-05	20	17	0.06864
WT RER	4.38E-06	1.09E-06	22	15	0.06630
Kir6.2-/- RER	7.00E-05	4.00E-05	20	17	0.05176
